# ESO-Det: An Efficient Small Object Detector for Real-Time UAV Perception

**DOI:** 10.3390/s26051512

**Published:** 2026-02-27

**Authors:** Haodong Deng, Song Zhou, Weidong Yang

**Affiliations:** State Key Laboratory of Multispectral Information Intelligent Processing Technology, School of Artificial Intelligence and Automation, Huazhong University of Science and Technology, Wuhan 430074, China; denghd@hust.edu.cn (H.D.); songz@hust.edu.cn (S.Z.)

**Keywords:** feature fusion, real-time detection, small objects, UAV object detection

## Abstract

Object detection in aerial drone imagery has attracted increasing attention in Unmanned Aerial Vehicle(UAV) sensing applications. However, small objects occupying limited image regions, with large scale variations and similar background interference, make it challenging to perceive them. Meanwhile, the constrained computing power of the onboard platform imposes requirements on the speed and efficiency of the algorithm. In this paper, we propose an efficient object detection network for real-time UAV perception named ESO-Det. Our approach introduces three key innovations: (1) Dense Cross-branch Complementary Module, a lightweight model that dynamically integrates semantic and spatial information to improve the network’s understanding of scene details. (2) Large-Kernel Context Integration Module, a module that expands receptive fields to effectively aggregate multi-scale contextual information. (3) Lightweight Selective Aggregation Module, a model selectively aggregates fused multi-scale features through different functional branches. Extensive experiments demonstrate that the proposed method achieves higher performance than representative existing approaches while maintaining real-time processing capability. The results show that our method is suitable for real-time UAV object detection.

## 1. Introduction

Unmanned aerial vehicles (UAVs) provide flexible data acquisition and offer unique perspectives, enhancing our ability to perceive scenes more comprehensively. As UAV technology continues to develop, UAVs equipped with cameras are now widely used in various fields, including fire safety, smart traffic management, and military reconnaissance [1,2,3]. However, object detection-based UAV scene understanding algorithms still face numerous challenges.

Existing object detection methods can be categorized into Transformer-based and CNN-based approaches [4]. Transformer-based detection networks use self-attention to model global relationships, achieving strong performance on benchmarks like MS COCO [5]. However, they often rely on low-resolution feature maps and have high computational costs due to the quadratic scaling of attention operations. This limits their real-time deployment on edge devices and makes them less effective in detecting small, dense objects and weak textures in UAV imagery [6]. CNN-based networks, typically represented by You Look Only Once (YOLO) [7], treat object detection as a classification and regression task, offering real-time performance with a focus on local context. These models are primarily designed for general object in ground-level scenarios. When applied to UAV imagery with small objects and complex backgrounds, their performance drops significantly [8]. Recently, several studies have focused on small objects. Some methods reduce the detection regions via clustering operations and dynamically calculate sampling points and offsets by introducing deformable convolutions [9,10]. These methods have achieved promising performance, yet they incur additional computational costs. Other studies focus their optimization efforts on key components such as the network’s feature extraction backbones and detection classification heads [11,12]. Although some achievements have been made, there remains significant room for improvement in UAV scenarios.

Despite the maturity of object detection algorithms, UAV image object detection still faces several main challenges. UAV images often cover wide fields of view, where targets occupy only a small fraction of the scene, making feature representation difficult. In addition, aerial perspectives introduce large scale variations, complicating multi-scale feature representation. Moreover, real-world UAV applications impose strict real-time requirements under limited onboard computational resources, making it challenging to balance accuracy and efficiency.

To address these challenges, we propose a detection network named ESO-Det, designed with a holistic approach that optimizes the entire detection pipeline. Our model is built upon the YOLO11 [13] baseline, which provides a strong and efficient foundation for real-time UAV object detection. We analyze the characteristics of the entire feature extraction and fusion process, design three core modules and a network structure in a targeted manner. We also adopt lightweight design strategies effectively to construct our method.

During the backbone feature extraction stage, as the resolution of feature maps decreases, spatial information weakens, semantic information strengthens, and small object details are progressively lost [12]. We propose the Dense Cross-branch Complementary Module (DCCM). Unlike conventional spatial–semantic complementary modules that rely on partial feature reuse, DCCM adopts a full-feature reparameterized design to extract richer representations during training while maintaining inference efficiency. The dual branches are dynamically fused through bidirectional calibration rather than simple concatenation, enabling adaptive emphasis on spatial or semantic cues according to scene characteristics.

As the resolution of feature maps decreases, multiple max-pooling operations lead to the loss of fine-grained contextual information [14], especially in small object detection tasks. To address this, we introduce the Large-Kernel Context Integration Module (LKCIM), which uses large-kernel receptive fields to aggregate multi-scale contextual information. This preserves important details and enables more effective integration of information from different receptive fields. LKCIM employs continuous strip-based large-kernel modeling to expand receptive fields without introducing gridding artifacts or quadratic complexity. Positioned before the final global attention layer, it progressively enlarges contextual perception, enabling a staged enhancement from local semantics to global awareness.

In the feature pyramid network, changes in feature sampling dimensions can introduce redundant information during fusion, making it difficult to extract effective features [15]. To address this, we propose the Lightweight Selective Aggregation Module (LSAM), which decouples multi-scale features into parallel branches, each focusing on specific feature types, enabling selective enhancement and reducing background interference. Compared to CSP-style feature partitioning, LSAM extends beyond simple feature splitting and reuse. It performs structured functional decomposition on fused features, where complementary branches refine representation diversity before unified integration, improving efficiency-aware multi-scale modeling.

Unlike approaches that introduce entirely new primitive operators, our work focuses on a UAV-oriented structural redesign of the detection pipeline. Instead of proposing isolated architectural components, we reorganize feature extraction, contextual modeling, and feature aggregation stages in a coordinated manner. This design emphasizes structural synergy and efficiency-aware integration rather than standalone module invention. Our key innovations are as follows:

- We propose a streamlined architecture for small object detection in UAV scenarios, utilizing high-resolution feature layers in the FPN to enhance detection performance;
- We introduce a comprehensive detection model optimized across the entire pipeline, consisting of three core modules. DCCM and LKCIM enhances backbone feature extraction, while LSAM efficiently handles feature fusion. These modules work synergistically, enabling our algorithm to achieve optimal performance;
- Extensive experiments on mainstream UAV benchmarks demonstrate notable improvements in detection accuracy, while maintaining real-time inference speed suitable for UAV deployment.

## 2. Related Works

### 2.1. Real-Time Object Detectors

The evolution of real-time object detection has consistently centered on the trade-off between accuracy and speed. Early two-stage detectors, represented by models such as Faster R-CNN [16], typically suffer from high inference latency due to their two-stage pipeline and complex region proposal mechanism, making them challenging to deploy in real-time applications. The emergence of single-stage detectors, such as YOLO and Single Shot Multibox Detector [17], revolutionized object detection by framing it as a dense grid regression task, significantly improving inference speed. The YOLO series has continually evolved with engineering-oriented optimizations. Subsequent versions introduced key innovations such as multi-scale predictions via Feature Pyramid Networks [15,18], the use of efficient backbones like CSPDarknet53 [19,20], and reparameterized convolutional architectures from RepVGG [21] to improve computational efficiency [22]. Latest improvements focus on detection heads to enhance both classification and localization accuracy [23], and the introduction of a global self-attention mechanism to refine feature representation [13].

Recently, transformer-based detection frameworks like DETR [24] introduced an end-to-end detection paradigm by eliminating anchor boxes and Non-Maximum Suppression, but suffer from high computational complexity. Later, RT-DETR [25] and D-FINE [26] focus on architectural optimization and refined regression modeling, enabling more practical deployment with improved inference efficiency. Despite the strong global context modeling ability of Transformer-based detectors, their high training cost and limited efficiency on embedded platforms remain challenging.

### 2.2. Small Object Detectors

Early UAV object detection methods mainly followed a coarse-to-fine paradigm. ClusDet and DMNet [27,28] adopted clustering strategies and density estimation to guide region cropping and re-detection. These approaches effectively handled large scale variations, but relied on additional preprocessing steps. Later studies shifted the focus toward internal feature modeling. GLSAN [9] introduced lightweight attention to enhance global context perception, while CESAC [13] improved receptive field flexibility through adaptive convolution in the detection head. More recent methods further explored feature interaction mechanisms for UAV object detection. YOLC [10] enhanced detection performance by strengthening feature interaction. FBRT-YOLO [12] focused on complementary feature modeling in semantic space to improve feature representation during feature extraction. In this paper, we focus on the entire detection pipeline and design an efficient network by jointly considering feature extraction and feature fusion.

## 3. Proposed Method

### 3.1. Architecture Overview

The proposed method is built upon three core modules which are designed to improve feature representation across the detection pipeline. The DCCM enhances backbone feature extraction by jointly modeling spatial cues and semantic information, enabling more robust representation of small objects. The LKCIM strengthens contextual modeling in deep feature layers by expanding the receptive field, allowing the network to effectively capture extended surrounding context. The LSAM refines multi-scale feature aggregation in the feature pyramid network by selectively emphasizing informative features while suppressing redundancy.

**Figure 1:**
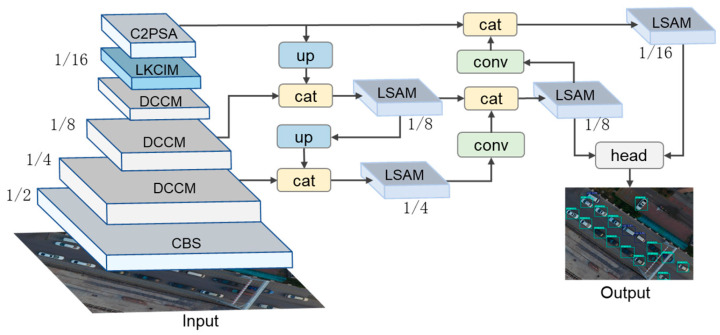
Overall architecture of the proposed ESO-Det.

In addition, we also apply a design on the baseline [12]. A high-resolution feature layer is introduced in the FPN to preserve small-object details, while the detection head is simplified to maintain efficient inference. The overall network structure is shown in Figure 1.

### 3.2. Dense Cross-Branch Complementary Module

**Figure 2:**
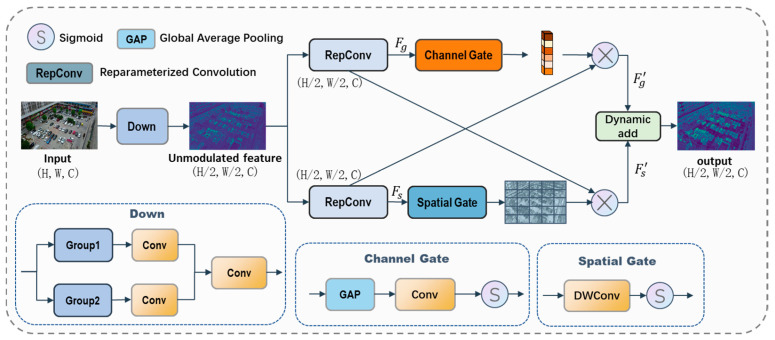
Architecture diagram of DCCM. DWConv denotes depth-wise convolution. Group means equal grouping along the channel dimension. Dynamic add represents dynamic weighted fusion of features.

In UAV scenes, small objects are often globally sparse but locally clustered, which requires effective integration of spatial localization cues and semantic context [12]. However, CSP-style [29] backbone blocks mainly focus on efficiency through feature splitting and partial reuse, where spatial and semantic information are still mixed implicitly within a unified feature stream. Here, we propose DCCM, which explicitly models spatial and semantic information through a dual-branch architecture as shown in Figure 2.

DCCM consists of a spatial branch and a semantic branch, each focusing on complementary aspects of feature representation. To fully exploit the complementary characteristics of the two feature streams, both branches adopt a reparameterized design [21] during feature extraction. The multi-branch structure is fully optimized during training and merged into an equivalent single branch during inference, enabling richer feature representation than conventional single-path convolution. Then, the spatial branch generates a spatial attention mask to highlight target regions and suppress background interference, whereas the semantic branch produces channel-wise weights to enhance discriminative features. This bidirectional calibration process can be formulated as follows:

(1) $$\left\{\begin{array}{l} F_{g}^{\prime }=F_{g}⊗f_{sp}(F_{s})\\ F_{s}^{\prime }=F_{s}⊗f_{ch}(F_{g}) \end{array}\right.$$

where $F_{s}$ and $F_{g}$ denote the spatial and semantic features, respectively. ⊗ denotes element-wise multiplication. $f_{sp}(\cdot )$ is the spatial gate consists of a depthwise convolution and a sigmoid function. $f_{ch}(\cdot )$ denotes channel gate consists of a global average pooling, a 1 × 1 convolution and a sigmoid function. Through this design, spatial encoding and channel-wise transformation are jointly performed to enhance feature representation.

Finally, the bidirectionally calibrated spatial and semantic features are fused through learnable weighted summation to obtain the output representation:

(2) $$F_{out}=\alpha F_{s}^{\prime }+(1-\alpha )F_{g}^{\prime }$$

where α is a learnable scalar coefficient introduced to control the relative contribution of spatial localization cues and semantic context as the feature maps go deeper. It is initialized to 0.5 and jointly optimized during training. The same α is used within each DCCM block, and it is independent across layers. This fusion yields complementary feature which improves localization accuracy and robustness by fully integrating semantic and spatial coding information.

### 3.3. Large-Kernel Context Integration Module

**Figure 3:**
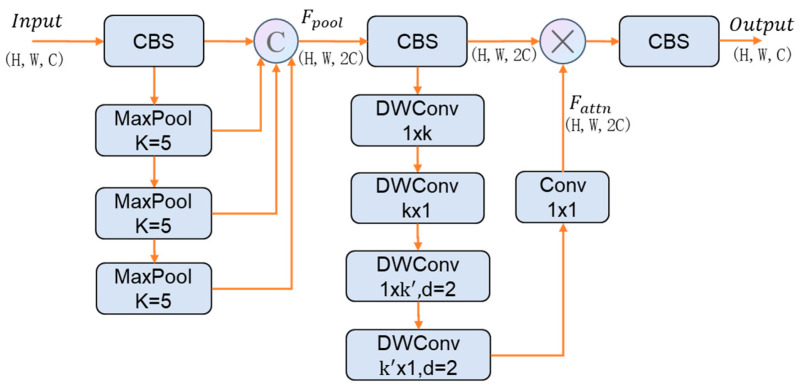
Architecture diagram of LKCIM. CBS denotes convolution, batch normalization, and SiLU activation. DWConv denotes depth-wise convolution.

As the backbone deepens, max pooling is commonly adopted to aggregate information from different receptive fields, providing coarse contextual cues. This process is static and lacks learnable parameters, making it difficult to selectively emphasize informative regions in complex scenes [14]. As shown in Figure 3, LKCIM enhances contextual perception across different receptive fields by introducing an enlarged spatial receptive attentive mechanism [30].

Firstly, it reduces the channel dimension of the input features to obtain compressed features to lowers computational load while filtering redundant information. Then, a series of max pooling layers with kernel size k = 5 are applied sequentially, producing four groups of features with progressively increasing receptive fields. Different receptive fields help with capturing objects at different scales. These four feature groups are concatenated along the channel dimension to form the multi scale fused feature $F_{pool}$ as follows:

(3) $$F_{pool}=\mathrm{Concat}(F^{\prime },\;f_{mp}^{1}(F^{\prime }),\;f_{mp}^{2}(F^{\prime }),\;f_{mp}^{3}(F^{\prime }))$$

where $f_{mp}$ denotes a max pooling operation with a kernel size of 5 × 5 and $f_{mp}^{i}(\cdot )$ represents i successive applications of this operation.

The feature is normalized via batch normalization to stabilize feature distribution and accelerate network convergence. To expand the context perception range of the convolution process, we adopt large-sized convolution kernels. To avoid the computational overhead caused by the direct use of large kernels, the features are processed through a sequential structure consisting of a standard large kernel separable strip convolution and a dilated separable strip convolution. This design captures both local spatial structures and wider contextual dependencies. Finally, a pointwise convolution restores the channel dimension to produce the enhanced output feature. As formulated below:

(4) $$F_{attn}=F_{pool}⊗{\mathrm{Conv}}_{1\times 1}(\varphi (F_{pool}))$$

(5) $$F_{attn}\;=\;{\mathrm{Conv}}_{1\times 1}(F_{attn})$$

where φ(⋅) denotes the large-kernel separable attention composed of a standard strip convolution and a dilated strip convolution. The attention map $F_{attn}$ is generated through a combination of standard and depthwise separable strip large-kernel convolutions. This design enhances the network’s positional sensitivity over a larger receptive field via bidirectional convolutions [31].

### 3.4. Lightweight Selective Aggregation Module

**Figure 4:**
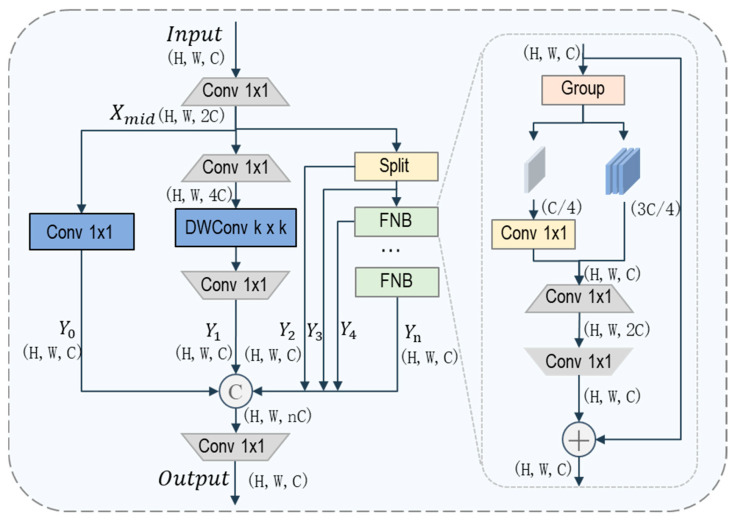
Architecture diagram of LSAM. Trapezoidal modules indicate changes in the channel dimension, while rectangular modules indicate no change in the channel dimension. DWConv denotes depthwise convolution, and FNB is the abbreviation for Faster Net Block. Group denotes grouping along the channel dimension.

Before feeding into the detection head, features undergo cross-interaction between high and low-resolution features via PANet [32] to fully extract discriminative key features. Although this process aligns features across different scales, the fused representations often mix heterogeneous information with varying importance. Such feature coupling may introduce redundancy and background interference, especially in scenarios where targets are sparsely distributed. Thus, we propose LSAM to refine fused multi-scale features through parallel feature processing. As shown in Figure 4, LSAM decouples fused features into multiple branches, where each branch focuses on a specific type of feature modeling. This design enables selective enhancement while suppressing background interference in complex scenes.

LSAM first performs dimensional recalibration on the input features, producing the intermediate features. Then, the features are decomposed into multiple parallel branches, each responsible for modeling complementary aspects of the fused representation [33]. Specifically, a channel calibration branch emphasizes core semantic information through channel-wise recalibration, while a lightweight spatial encoding branch captures spatial structures and fine-grained details with limited computational overhead. In addition, a feature preservation branch directly forwards part of the intermediate features to avoid information loss caused by repeated transformations. Finally, a dynamic enhancement branch strengthens contextual representations under occlusion and background clutter using efficient feature modulation. The process can be expressed as:

(6) $$\left\{Y_{0},\;Y_{1},\;Y_{2},\;\ldots ,Y_{n}\right\}=\delta (X_{\mathrm{mid}})$$

where δ(⋅) denotes the multi-branch feature splitting operation, including channel calibration, lightweight spatial encoding, feature preservation, and a dynamic enhancement branch. Redundant computation exists in the dynamic enhancement branch itself. FasterNet Blocks (FNB) [34] are adopted to compress computational overhead during module stacking via partial convolution to boost efficiency. This design is well suited for addressing feature redundancy.

The branch decomposition is structural rather than weighted selection, and adaptive modulation is introduced only within the dynamic enhancement branch. All branches are concatenated and compressed using a 1 × 1 convolution to produce the final output feature as follows:

(7) $$X_{\mathrm{out}}={\mathrm{Conv}}_{1\times 1}(\mathrm{Concat}(Y_{0},Y_{1},\ldots ,Y_{i}))$$

## 4. Experimental Results

### 4.1. Datasets

To evaluate the effectiveness and generalization ability of our method, experiments are conducted on two widely used UAV object detection benchmarks:

VisDrone2019 [35], which is a large-scale UAV dataset covering diverse scenes and imaging conditions. Its object detection subset contains multiple categories such as pedestrians and vehicles. Most targets appearing at small scales and in densely distributed scenes, thus it is suitable for evaluating small object detection performance.

UAVDT [36] focuses on vehicle detection from UAV perspectives and is characterized by dense target distributions, frequent occlusions, and complex backgrounds. This dataset is used to test the robustness of the proposed method under challenging UAV scenarios.

### 4.2. Implementation Details

The experiments are conducted on an Intel i9-13900K CPU (Intel Corporation, Santa Clara, CA, USA), with an NVIDIA RTX 3090 GPU and 32 GB of system memory (Nvidia Corporation, Santa Clara, CA, USA). The key training parameters are as follows: initial learning rate was 0.01 and dynamically adjusted using a cosine annealing scheduler, batch size was 16, training epochs were 300, the optimizer selected was SGD with a momentum of 0.937 and weight decay of 0.0005. Mosaic data augmentation is applied throughout the training stage. The main environment was implemented using Python 3.11, Ultralytics 8.3.9, CUDA 12.1, cuDNN 9.1.0 and PyTorch 2.5.1, with NumPy 1.26.4.

For evaluation, the platform consisted of an Intel i5-13500HX CPU (Intel Corporation, Santa Clara, CA, USA) and an NVIDIA RTX 4060 Laptop GPU (Nvidia Corporation, Santa Clara, CA, USA). Evaluation was conducted using the PyTorch framework, with the model running in FP16 mixed precision. The input resolution was set to 640 × 640 during evaluation. The intersection over union (IoU) threshold for non-maximum suppression is set to 0.7. Test-time augmentation was disabled, and all latency measurements were obtained under a batch size of 1 to reflect practical online deployment conditions to closely simulate real-time online image processing. Latency includes the total elapsed time for preprocessing, inference, and postprocessing.

### 4.3. Evaluation Metrics

This study adopts widely used evaluation metrics in object detection to assess performance from three aspects: detection accuracy, real-time inference capability, and model efficiency. The specific metrics include:

- Average Precision (AP), covering overall performance and per category accuracy, to quantify core detection capability.
- Average Recall (AR), evaluating the model’s sensitivity across IoU thresholds and object scales, to reflect the completeness of target instance retrieval.
- Number of Parameters (Params) and Computational Complexity (GFLOPs), evaluating the model’s lightweight performance and hardware compatibility.
- Latency, measuring the total elapsed time for preprocess, inference, and postprocess to evaluate real time efficiency and feasibility for onboard deployment.

### 4.4. Ablation Study

To analyze the contribution of each proposed component, ablation experiments are conducted on the VisDrone2019 dataset. The experiments progressively introduce the proposed modules to evaluate their individual and cumulative effects on detection performance, model complexity, computational cost and Latency. The baseline model is trained using the official training recipe with default hyperparameters for visual object detection, as described in Section 4.2. The implementation follows the official open-source code and configuration of YOLO11.

As shown in Table 1, using only the lightweight architecture (LA) significantly reduces model complexity while maintaining comparable accuracy. When adding DCCM, AP_50_ improvement of more than 0.9% indicates a clear enhancement in the model’s target recognition capability. Thanks to the reparameterized design, the training-stage computational complexity is slightly increased, while branch fusion during inference enables richer learned information and more efficient inference. Further incorporating LKCIM leads to further improvements in detection accuracy. The full integration of all modules achieves the best result with an AP of 21.06%, while parameters remain low at 0.84 M and FLOPs at 6.8 G. This result shows that our method remains competitive on accuracy and efficiency.

Individual module ablation results indicate that all newly introduced modules independently improve detection performance without significantly increasing computational cost and DCCM improves accuracy while maintaining comparable latency. This demonstrates that the proposed design effectively balances detection accuracy with inference speed, making it well-suited for the computational capabilities and real-time requirements of UAV platforms.

Table 2 presents the scale-wise evaluation results. AR_S_, AR_m_, and AR_l_ denote the average recall computed over IoU thresholds from 0.5 to 0.95 for small, medium, and large objects, following the COCO evaluation protocol.

After introducing DCCM, a modest improvement is observed for small objects (AP_50s_: +0.3%, ARs: +0.2%), while medium- and large-scale recall exhibit slight fluctuations. This suggests that DCCM primarily benefits small-scale targets by reinforcing spatial–semantic complementarity, which helps mitigate feature attenuation in deeper layers. With the addition of LKCIM, performance improves across all object scales, particularly for medium-scale objects (AP_50m_: +1.5%). This indicates that expanded receptive fields enhance contextual perception and improve robustness across varying object sizes. Finally, incorporating LSAM yields consistent improvements on all scales in the full model, with balanced gains in both precision and recall. This suggests that selective branch-based processing of fused features refines multi-scale representations in a coordinated manner.

### 4.5. Contrast Experiments

In this section, we compare ESO-Det with several representative object detection approaches on UAV benchmarks to comprehensively evaluate the effectiveness of the proposed method. The selected methods cover different design paradigms and are widely used in UAV or real-time object detection scenarios. Specifically, we include lightweight single-stage detectors to assess real-time performance under constrained computational budgets, as well as recent UAV-oriented methods that are specifically designed to handle small objects. These approaches represent the current mainstream solutions for UAV object detection and provide a fair basis for comparison in terms of both accuracy and efficiency.

#### 4.5.1. Overall Comparison Results

To validate the performance advantages of the proposed algorithm, our proposed model is compared with several mainstream real-time object detection methods on the VisDrone2019 dataset, focusing on three key metrics: AP_50_ (emphasizing recall), AP_75_ (emphasizing localization precision), and AP (balancing precision and recall). The results are presented in Table 3.

All comparative experiments were conducted under the unified inference environment specified in Section 4.2, ensuring the fairness and validity of the performance comparison. The latency includes the full process of image preprocessing, model inference and postprocessing, ensuring the comparability and practical reference value of the latency results.

The comparison shows that compared to general real-time detectors such as the newest YOLO series and NanoDet [37], our algorithm achieves significantly higher detection accuracy in AP, AP_50_ and AP_75_ with a more lightweight design (0.8 M parameters, 6.8 G FLOPs). Notably, it outperforms Hyper-YOLO (3.6 M parameters, 9.5 G FLOPs) with only one-fourth of its parameters and lower latency, while achieving higher AP and AP_50_. This performance gain is attributed to semantic-spatial complementary design and large-kernel contextual modeling, both effectively boosting small object recognition in UAV scenes. When compared to the UAV-specialized model FBRT-YOLO, while both models exhibit similar parameter counts, computational costs, and real-time performance, our method achieves a 1.3% higher AP50, validating the effectiveness of our end-to-end pipeline design. In summary, ESO-Det successfully balances real-time capability and detection accuracy, offering an optimized solution for UAV onboard deployment.

#### 4.5.2. Performance Across Object Scales

**Figure 5:**
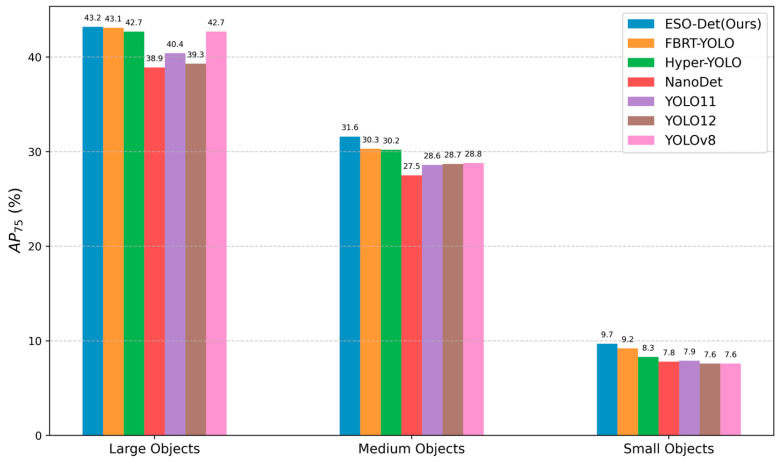
Performance across different object scales on AP_75_.

As illustrated in Figure 5, we evaluate the performance of the proposed method across different object scales, where small, medium, and large objects are defined according to the COCO standard [4]. The proposed method outperforms FBRT-YOLO by 1.3% on small objects, which is specifically designed for small-object scenarios. Notably, our method also gained a 0.5% performance improvement on medium objects. On large objects, our method attains a slight advantage. These results demonstrate robust detection performance across object scales, with particularly strong gains on small and medium objects that are more prevalent in UAV scenes.

#### 4.5.3. Performance Across Categories

**Figure 6:**
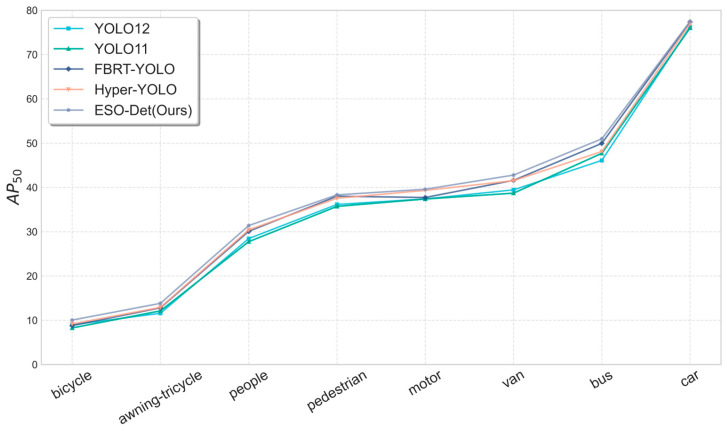
Performance across different object categories on AP_50_.

To evaluate per-category detection performance, we conducted a category-specific experiment on the eight core object categories in VisDrone2019. The per-category AP results are shown in Figure 6. Our method achieves the best performance in 7 out of the 8 categories. Compared to baseline, our method improves by 1.56% on the small-scale target pedestrian, and notably increases by 4.07% on the large-scale category bus. This demonstrates the algorithm’s ability to simultaneously capture features of tiny objects and also keeps modeling the structure of large ones. Although our method performs closely on pedestrian and motor with some other methods, it maintains a leading position in most categories and remains consistently among the top performers overall. With higher accuracy and more consistent activation patterns across all categories, our method fully demonstrates its robustness in handling different categories especially small objects from UAV perspectives.

#### 4.5.4. Results on UAVDT

Extended experiments are conducted on the UAVDT dataset to validate our method’s generalization ability. The results are presented in Table 4. Our proposed method comprehensively outperforms mainstream comparison algorithms such as GLSAN, CEASC, and FBRT-YOLO. It achieves an average precision (AP) of 19.1%, ranking second only to YOLC (19.3%). In terms of localization accuracy measured by AP75, it reaches 20.3%, surpassing all compared methods. The AP_50_ metric further improves to 31.8% which is also the highest performance. Results indicate that our proposed algorithm exhibits strong generalization ability for object detection in typical UAV scenarios.

### 4.6. Visualization Experiments

**Figure 7:**
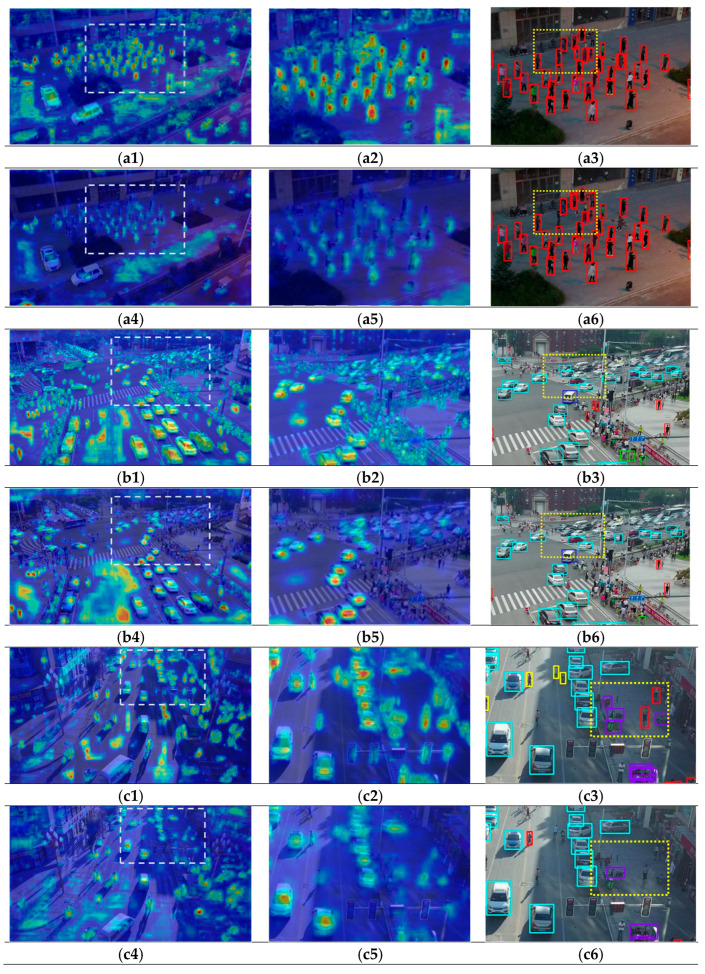
Visualization comparison of heatmaps and results between our method and baseline. (**a**–**c**) represent three typical UAV tracking scenarios, where (**a**) denotes dense small targets, (**b**) drastic large scale variations, and (**c**) complex background environments. **1**–**3** denote the global heatmap, local heatmap and local detection results of the proposed method, while **4**–**6** represent the three corresponding results of the baseline method. The images in the second column show the regions marked by white boxes in the first column. The yellow dashed boxes indicate regions with obvious differences between the baseline and our method.

To visually compare the performance of the algorithms in UAV scenarios, we selected three typical scenes for a visualization experiment. We compare the feature heatmaps from the input head of the baseline model and ESO-Det. By applying gradient based class activation mapping to project intermediate features onto the image plane, the resulting feature maps reflect the model’s perceptual intensity toward targets of different scales and receptive fields. The results are shown in Figure 7.

In small object and multi scale dense scenes, the baseline model shows a clear preference for large scale targets and exhibits weaker response around object centers. In contrast, ESO-Det activates different target regions more evenly and focuses more precisely on the objects themselves. Under challenging illumination conditions such as strong light or shadows, ESO-Det demonstrates stronger robustness in distinguishing foreground from background. It maintains stable attention on targets even in high contrast areas, which helps improve detection stability and localization accuracy.

## 5. Discussion

The experimental results show that our method consistently improves detection performance in UAV scenarios, particularly for small objects. The final model contains only 0.84 M parameters and requires 6.8 GFLOPs, which is substantially lower than many recent UAV-oriented detectors. Moreover, the architecture avoids dynamic convolution, deformable convolution, and heavy attention mechanisms, relying instead on standard convolutional operators that are well supported by existing embedded inference engines.

Many recent detection methods improve performance through additional preprocessing or specialized modules, which may reduce hardware efficiency due to irregular memory access patterns. Although effective, these approaches often increase model complexity or rely on additional processing steps. Our improvements can be attributed to the joint optimization of feature representation and multi-scale perception across the detection pipeline, rather than focusing on a single network component.

We consider feature extraction, contextual modeling, and feature aggregation in a coordinated manner across the detection pipeline. In the backbone, dynamic spatial–semantic calibration with a reparameterized design alleviates feature attenuation for small objects while preserving inference efficiency. Although introducing DCCM slightly increases FLOPs, it does not lead to higher inference latency, since its multi-branch structure is reparameterized into an equivalent single-path convolution during inference. As a result, no additional runtime operators are introduced, preserving hardware efficiency. At deeper semantic stages, large-kernel strip modeling expands receptive fields in a computationally efficient way, progressively enhancing contextual perception. In the neck, high-resolution features are retained, and fused representations are structurally decomposed rather than simply reused, enabling complementary refinement beyond CSP-style partitioning. This pipeline-level structural coordination, combined with lightweight operator choices, contributes to a favorable trade-off between detection accuracy and computational cost in UAV scenarios.

Although our modules share high-level motivations with existing patterns, their design and integration differ in key aspects. DCCM follows a dual-branch complementary paradigm but distinguishes itself through reparameterized training–inference decoupling and dynamic bidirectional fusion, rather than static concatenation. LKCIM is integrated on multi-scale pooled features before the global attention stage, forming staged contextual enhancement instead of acting as an isolated attention block. LSAM introduces functional decomposition of fused features, differing from CSP-style partial reuse strategies. These distinctions highlight pipeline-level coordination rather than direct module substitution.

While our model achieves favorable performance on small object detection tasks, challenges remain in extremely dense UAV scenes with severe object occlusion. In such condition, object textures become increasingly weak and are often represented by only limited visual features. Moreover, object overlap can disrupt bounding box assignment, particularly in scenes dominated by small objects. Future work will explore multi-task learning strategies to alleviate these limitations. For instance, integrating lightweight object tracking or fine-grained segmentation cues could provide complementary temporal or structural information. In particular, tracking-based approaches may leverage multi-frame temporal consistency to stabilize detection results and reduce performance degradation under severe occlusion. Furthermore, future research will investigate model optimization and validation on embedded UAV platforms to evaluate practical deployment feasibility.

## 6. Conclusions

In this paper, we propose an efficient small object detection framework ESO-Det for real-time UAV perception tasks. We analyze the root causes of small-object feature degradation during deep network feature propagation, including cross-branch semantic-spatial information mismatch, insufficient contextual aggregation of small targets, and redundant multi-scale feature fusion. To address these issues in a targeted manner, we design three lightweight modules (DCCM, LKCIM, LSAM) to optimize backbone feature extraction, deep-layer contextual modeling and FPN multi-scale feature aggregation respectively. We also adopt a streamlined network architecture with high-resolution feature retention. This end-to-end pipeline optimization effectively enhances the model’s feature representation and small target recognition capability for UAV scenes with large scale variations and complex backgrounds. Extensive experiments on VisDrone2019 and UAVDT show our method outperforms state-of-the-art real-time lightweight detectors for UAV perception, with an ultra-lightweight architecture and real-time inference latency under 12 ms per frame, indicating its potential for UAV onboard deployment.

## Figures and Tables

**Table 1:** Ablation study performance comparison on Visdrone2019.

LA	DCCM	LKCIM	LSAM	AP (%)	AP_50_ (%)	Params (M)	FLOPs (G)	Latency (ms)
				19.46	33.40	2.58	6.3	11.5
√				19.78	33.04	0.71	**5.3**	10.2
√	√			19.80	33.98	**0.69**	5.8	**9.2**
√		√		19.57	33.47	0.78	5.5	10.0
√			√	20.46	34.92	0.80	6.1	11.1
√	√	√		20.34	34.57	0.76	6.0	9.6
√	√		√	21.03	35.26	0.78	6.7	10.5
√		√	√	20.67	35.18	0.87	6.4	10.7
√	√	√	√	**21.06**	**35.67**	0.84	6.8	11.0

√ indicates the corresponding module is added. All items without √ represent the baseline. Bold values indicate the optimal performance among the comparison methods.

**Table 2:** Scale-wise evaluation on Visdrone2019.

LA	DCCM	LKCIM	LSAM	AP_50s_ (%)	AP_50m_ (%)	AP_50l_ (%)	AR_S_ (%)	AR_m_ (%)	AR_l_ (%)
√				22.0	46.3	33.04	21.8	46.4	54.1
√	√			22.3	46.5	33.98	22.0	46.1	52.9
√	√	√		22.5	48.0	34.57	22.2	47.4	53.5
√	√	√	√	**23.2**	**49.3**	**35.67**	**23.1**	**48.6**	**56.7**

√ indicates the corresponding module is added. All items without √ represent the baseline. Bold values indicate the optimal performance among the comparison methods.

**Table 3:** Basic performance comparison on VisDrone2019.

Method	Params (M)	FLOPs (G)	AP (%)	AP_75_ (%)	AP_50_ (%)	Latency (ms)
NanoDet	2.0	**2.8**	18.6	17.9	33.9	13.8
YOLOv8	3.1	8.0	19.5	19.5	33.3	**9.2**
YOLO11	2.6	6.3	19.6	19.6	33.4	11.5
YOLO12	2.6	6.3	19.2	19.3	32.9	16.0
FBRT-YOLO [12]	0.9	6.7	20.2	20.8	34.4	10.9
Hyper-YOLO	3.6	9.5	20.6	20.0	35.2	12.7
ESO-Det (Ours)	**0.8**	6.8	**21.1**	**21.0**	**35.7**	11.0

Bold values indicate the optimal performance among the comparison methods.

**Table 4:** Basic performance comparison on UAVDT.

Method	AP (%)	AP_75_ (%)	AP_50_ (%)
GLSAN [9]	17.0	18.8	28.1
GFL [38]	16.9	17.9	29.5
CEASC [11]	17.1	17.8	30.9
YOLC [10]	**19.3**	20.1	30.9
FBRT-YOLO [12]	18.4	19.4	31.1
ESO-Det (Ours)	19.1	**20.3**	**31.8**

Bold values indicate the optimal performance among the comparison methods.

## Data Availability

The data presented in this study are available upon request from the author.

## References

[1] Tahir M.A., Mir I., Islam T.U. (2023). A Review of UAV Platforms for Autonomous Applications: Comprehensive Analysis and Future Directions. IEEE Access.

[2] Chen X., Li Y., Zhang W. Vision-Based UAV Systems for Traffic Surveillance and Emergency Response. Proceedings of the IEEE/CVF Conference on Computer Vision and Pattern Recognition.

[3] Wu X., Li W., Hong D., Tao R., Du Q. (2022). Deep Learning for Unmanned Aerial Vehicle-Based Object Detection and Tracking: A Survey. IEEE Geosci. Remote Sens. Mag..

[4] Arkin E., Yadikar N., Muhtar Y., Ubul K. A Survey of Object Detection Based on CNN and Transformer. Proceedings of the 2nd IEEE International Conference on Pattern Recognition and Machine Learning (PRML).

[5] Lin T.-Y., Maire M., Belongie S., Hays J., Perona P., Ramanan D., Dollár P., Zitnick C.L. Microsoft COCO: Common Objects in Context. Proceedings of the European Conference on Computer Vision (ECCV) 2014.

[6] Rekavandi A.M., Rashidi S., Boussaid F., Hoefs S., Akbas E., Bennamoun M. (2023). Transformers in Small Object Detection: A Benchmark and Survey of State-of-the-Art. ACM Comput. Surv..

[7] Redmon J., Divvala S.K., Girshick R.B., Farhadi A. You Only Look Once: Unified, Real-Time Object Detection. Proceedings of the 2016 IEEE Conference on Computer Vision and Pattern Recognition (CVPR).

[8] Ma C., Fu Y., Wang D., Guo R., Zhao X., Fang J. (2023). YOLO-UAV: Object Detection Method of Unmanned Aerial Vehicle Imagery Based on Efficient Multi-Scale Feature Fusion. IEEE Access.

[9] Deng S., Li S., Xie K., Song W., Liao X., Hao A., Qin H. (2021). A Global-Local Self-Adaptive Network for Drone-View Object Detection. IEEE Trans. Image Process..

[10] Liu C., Gao G., Huang Z., Hu Z., Liu Q., Wang Y. (2024). YOLC: You Only Look Clusters for Tiny Object Detection in Aerial Images. IEEE Trans. Intell. Transp. Syst..

[11] Du B., Huang Y., Chen J., Huang D. Adaptive Sparse Convolutional Networks with Global Context Enhancement for Faster Object Detection on Drone Images. Proceedings of the IEEE/CVF Conference on Computer Vision and Pattern Recognition.

[12] Xiao Y., Xu T., Xin Y., Li J. FBRT-YOLO: Faster and Better for Real-Time Aerial Image Detection. Proceedings of the AAAI Conference on Artificial Intelligence.

[13] Jocher G. YOLO11 by Ultralytics. https://github.com/ultralytics/ultralytics.

[14] Chen L.-C., Papandreou G., Kokkinos I., Murphy K., Yuille A.L. (2018). DeepLab: Semantic Image Segmentation with Deep Convolutional Nets, Atrous Convolution, and Fully Connected CRFs. IEEE Trans. Pattern Anal. Mach. Intell..

[15] Lin T.-Y., Dollár P., Girshick R., He K., Hariharan B., Belongie S. Feature Pyramid Networks for Object Detection. Proceedings of the IEEE Conference on Computer Vision and Pattern Recognition (CVPR).

[16] Ren S., He K., Girshick R., Sun J. (2016). Faster R-CNN: Towards Real-Time Object Detection with Region Proposal Networks. IEEE Trans. Pattern Anal. Mach. Intell..

[17] Liu W., Anguelov D., Erhan D., Szegedy C., Reed S., Fu C.Y., Berg A.C. (2016). SSD: Single Shot MultiBox Detector.

[18] Redmon J. YOLOv3 by Pjreddie. https://github.com/pjreddie/darknet.

[19] Bochkovskiy A. YOLOv4 Official Implementation. https://github.com/AlexeyAB/darknet.

[20] Jocher G. YOLOv5 by Ultralytics. https://github.com/ultralytics/yolov5.

[21] Ding X., Zhang X., Ma N., Han J., Ding G., Sun J. RepVGG: Making VGG-style ConvNets Great Again. Proceedings of the 2021 IEEE/CVF Conference on Computer Vision and Pattern Recognition (CVPR).

[22] Li C. YOLOv6 by Meituan. https://github.com/meituan/YOLOv6.

[23] Jocher G., Chaurasia A., Qiu J. Ultralytics/Ultralytics: NEW—YOLOv8. https://github.com/ultralytics/ultralytics.

[24] Carion N., Massa F., Synnaeve G., Usunier N., Kirillov A., Zagoruyko S. (2020). End-to-End Object Detection with Transformers. Proceedings of the European Conference on Computer Vision.

[25] Zhao Y., Lv W., Xu S., Wei J., Wang G., Dang Q., Liu Y., Chen J. Detrs beat yolos on real-time object detection. Proceedings of the IEEE/CVF Conference on Computer Vision and Pattern Recognition.

[26] Peng Y., Li H., Wu P., Zhang Y., Sun X., Wu F. D-FINE: Redefine Regression Task in DETRs as Fine-Grained Distribution Refinement. Proceedings of the 13th International Conference on Learning Representations (ICLR).

[27] Yang F., Fan H., Chu P., Zhang Y., Sun X., Wu F. Clustered Object Detection in Aerial Images. Proceedings of the IEEE/CVF International Conference on Computer Vision.

[28] Li C., Yang T., Zhu S., Chen C., Guan S. Density Map Guided Object Detection in Aerial Images. Proceedings of the IEEE/CVF Conference on Computer Vision and Pattern Recognition Workshops.

[29] Wang C.Y., Liao H.Y.M., Wu Y.H., Chen P.Y., Hsieh J.W., Yeh I.H.C. CSPNet: A New Backbone That Can Enhance Learning Capability of CNN. Proceedings of the IEEE/CVF Conference on Computer Vision and Pattern Recognition Workshops.

[30] Lau K.W., Po L.M., Rehman Y.A.U. (2024). Large Separable Kernel Attention: Rethinking the Large Kernel Attention Design in CNN. Expert Syst. Appl..

[31] Guo M., Lu C., Liu Z., Cheng M., Hu S. (2022). Visual attention network. Comput. Vis. Media.

[32] Liu S., Qi L., Qin H., Shi J., Jia J. Path Aggregation Network for Instance Segmentation. Proceedings of the 2018 IEEE/CVF Conference on Computer Vision and Pattern Recognition.

[33] Feng Y., Huang J., Du S., Ying S., Yong J.-H., Li Y., Ding G., Ji R., Gao Y. (2024). Hyper-YOLO: When Visual Object Detection Meets Hypergraph Computation. IEEE Trans. Pattern Anal. Mach. Intell..

[34] Chen J., Kao S., He H., Zhuo W., Wen S., Lee C.-H., Chan S.-H.G. Run, Don’t Walk: Chasing Higher FLOPS for Faster Neural Networks. Proceedings of the IEEE/CVF Conference on Computer Vision and Pattern Recognition.

[35] Du D., Zhu P., Wen L., Bian X., Lin H., Hu Q., Peng T., Zheng J., Wang X., Zhang Y. VisDrone-DET2019: The Vision Meets Drone Object Detection in Image Challenge Results. Proceedings of the 2019 IEEE/CVF International Conference on Computer Vision Workshop (ICCVW).

[36] Du D., Qi Y., Yu H., Yang Y., Duan K., Li G., Zhang W., Huang Q., Tian Q. The unmanned aerial vehicle benchmark: Object detection and tracking. Proceedings of the European Conference on Computer Vision (ECCV).

[37] Lyu R. (2021). NanoDet-Plus: Super Fast and High Accuracy Lightweight Anchor-Free Object Detection Model. https://github.com/RangiLyu/nanodet.

[38] Li X., Wang W., Wu L., Chen S., Hu X., Li J., Tang J., Yang J. (2020). Generalized Focal Loss: Learning Qualified and Distributed Bounding Boxes for Dense Object Detection. arXiv.

